# The Time for Fly Swatting

**Published:** 1915-11

**Authors:** 


### The Time for Fly Swatting.

It will be remembered that we have not favored literal fly swatting, partly because the possible results are infinitesimal in comparison with methods of exclusion, prevention of fomites, and capture by traps, adhesive paper, etc., partly because of possible danger to the swatter. With the advent of cold weather, however, flies outdoors either die or become dormant. They seek shelter, however, entering through the screens and temporarily open doors and windows, in much greater numbers relatively than during the summer. Yet, the actual number in any house is small and within the possibilities of even manual capture. Extirpation by swatting, under these circumstances becomes practical and its value is not measured by the actual number killed but by their potential progeny next spring.

